# Immunohistochemical expressionof sodium-dependent glucose transporter - 2 (SGLT-2) in clear cell renal carcinoma: possible prognostic implications

**DOI:** 10.1590/S1677-5538.IBJU.2018.0271

**Published:** 2019

**Authors:** Minoru Kobayashi, Toshitaka Uematsu, Yuumi Tokura, Kohei Takei, Kazumasa Sakamoto, Takahiro Narimatsu, Akinori Nukui, Takao Kamai

**Affiliations:** 1 Department of Urology, Utsunomiya Memorial Hospital, Tochigi, Japan; 2 Department of Urology, Dokkyo Medical University, Tochigi, Japan; 3 Department of Urology, Nasu Red Cross Hospital, Tochigi, Japan

**Keywords:** Immunohistochemistry, Glucose Transport Proteins, Facilitative, Carcinoma, Renal

## Abstract

**Purpose:**

Glucose is a major energy resource for tumor cell survival and growth, and its influx into cells is mainly carried out by facilitative glucose transporters (GLUTs). Sodium - dependent glucose transporters (SGLTs) have been highlighted as playing important roles in diabetic treatment. However, their potential roles in cancer remain unclear. We examined expression patterns of SGLTs in tumor tissues together with conventional pathological variables to determine prognostic significance in patients with renal cell carcinoma (RCC).

**Materials and Methods:**

Nephrectomy specimens were obtained from 68 patients. GLUT - 1, - 2 and SGLT - 1, - 2 expression in tumor and adjacent normal tissues were analyzed by immunohistochemical staining, and intensity was quantified using an image analyzer.

**Results:**

The four glucose transporters evaluated were broadly distributed in tumor tissues as well as throughout the normal parenchyma. There was no significant correlation between transporter expression and conventional pathological variables. However, increased SGLT - 2 expression was significantly associated with shorter overall survival (p < 0.01), regardless of metastatic status.

**Conclusions:**

We propose possible prognostic significance of SGLT - 2 expression in human RCC. Given that glucose is a major energy resource for tumor cells and that glucose transport is largely mediated by SGLT, SGLT - 2 may serve as a possible therapeutic target in RCC.

## INTRODUCTION

Clear cell carcinoma is the most common type of renal cell carcinoma (RCC). It is characterized by clear cytoplasmic cells containing glycogen and is surrounded by abundant vasculature (1). Glucose oxidation represents a major source of metabolic energy for tumor cells. However, oxidative metabolism in tumor cells is less efficient despite an increased consumption of glucose (2). Alternatively, tumor cells cover their energy requirements by anaerobic glycolysis known as the Warburg effect, which demands high glucose levels. To survive in such a hostile microenvironment, tumor cells up - regulate expression of membrane glucose transporters, such as glucose transporters (GLUTs) and sodium - dependent glucose transporters (SGLTs) (3). GLUTs use existing gradients in glucose concentration between external and internal sides of the plasma membrane to facilitate translocation, while SGLTs move sugars utilizing a sodium - electrochemical gradient (4).

Fourteen GLUTs have been identified, with tissue - specific expression patterns reflecting varied glucose demands in different tissues. The normal kidney has been shown to express GLUT - 1, GLUT - 2, GLUT - 5, and GLUT - 9. In the renal tubules, GLUT - 1 and GLUT - 2 transport glucose along its concentration gradient under normal condition (5). GLUT - 5 is the fructose - specific transporter and was shown to upregulate together with GLUT - 1 and GLUT - 2 in response to the intracellular concentration of glucose in diabetic hyperglycemic rat model (6). GLUT - 9 was shown to be involved in urate homeostasis by its secretion on the distal tubules (7). GLUT - 1 is widely expressed in tumors as well as normal tissues. Increased GLUT - 1 expression is associated with a poor prognosis in a variety of cancers and is often observed in poorly differentiated tumors (8). Although there have been several studies indicating the constitutive expression of GLUT - 1 in RCC (9-12), GLUT - 1 expression is unlikely to be associated with pathological parameters or survival (9-11), leaving the significance of GLUT - 1 expression undetermined in RCC. The recent study identified the role of GLUT - 2 in association with the von Hipple - Lindau (VHL) gene status in RCC (10).

Similar to GLUT - 1 and GLUT - 2, SGLT - 1 and SGLT - 2 induction is also used by tumor cells to enhance glucose uptake and glycolysis to obtain sufficient energy for sustaining expansive growth (13). However, limited data are available regarding SGLT expression in tumors. SGLT - 1 is expressed in cervical, ovarian, head and neck, colorectal, prostate, lung, and pancreatic cancers. The latter three cancer types also express SGLT - 2 (13-19). SGLT - 1 overexpression has been shown to be associated with patient survival in pancreatic cancer (15). Recently, inhibitors that specifically target SGLT - 2 were approved as antidiabetic drugs. Given the wide use of SGLT - 2 inhibitors, investigating the relationship between SGLT and cancer is a practical next step. To the best of our knowledge, SGLT expression in RCC has not been reported and remains to be elucidated. With above - mentioned backgrounds, we also highlighted GLUT - 1 for its undetermined prognostic role and GLUT - 2 for its possible role in RCC. In the present study, we investigated the correlation between pathological parameters and expression of glucose transporters including GLUT - 1, GLUT - 2, SGLT - 1, and SGLT - 2, and their prognostic implications in human RCC.

## MATERIALS AND METHODS

We studied 68 consecutive Japanese patients with mean age of 62.3 years who were diagnosed with clear cell RCC from 2005 to 2015. All patients underwent a CT and / or MRI for preoperative staging. The postoperative follow-up period ranged from 5.7 to 146 months (median: 43.9 months, interquartile range: 34.7 - 67.1 months). All patients underwent a nephrectomy. Interferon-α, sorafenib, or sunitinib were typically provided to patients with metastasis after nephrectomy. Fifteen patients had a known history of diabetes mellitus (DM). Tumor characteristics are displayed in Table-1. The histological classification and pathological grade were determined on the basis of the General Rules for Clinical and Pathological Studies on Renal Cell Carcinoma in Japan (20). Sarcomatoid components were included in the tumor tissue in three patients. The clinical tumor stage was determined according to the TNM classification.

**Table 1:** Patient and tumor characteristics.

Patients		n
	No. of patients	68
	Age (mean, range)	62.3 (40-84)
	Gender (male / female)	51 / 17
	Follow-up period (months) (median, range)	43.9 (5.7-146)
**Tumor**		
	pT stage (T1 / T2 / T3 / T4)	32 / 13 / 21 / 2
	Histological grade (G1/G2/G3)	5 / 48 / 10
	Vascular invasion (v0 / v1)	31 / 37
	pN status (N0 / N1, 2)	61 / 7
	Metastasis (M0 / M1)	48 / 20

This study was conducted in accordance with the Helsinki Declaration and was approved by the ethical review board of Dokkyo Medical University Hospital. Each patient signed a consent form that was approved by our institutional Committee on Human Rights in Research.

### 

#### Immunohistochemistry and staining evaluation

Immunostaining was performed using the Histofine SAB - PO kit (Nichirei Biosciences Inc, Tokyo, Japan) according to manufacturer's instructions. Briefly, after sections (μm slices) were dewaxed and dehydrated, antigen retrieval was performed in citrate buffer (pH 6.0) in microwave for 5 minutes x 3 times for GLUT - 1 and GLUT - 2 staining, in autoclave at 125°C for 5 minutes for SGLT - 1 staining, or in water bath at 95°C for 40 minutes for SGLT - 2 staining. Then, sections were treated with 0.3% H_2_O_2_ in methanol and incubated with anti - GLUT - 1 (Abcam #ab115730, monoclonal, at 1 / 500 dilution), anti - GLUT - 2 (Abcam #ab85715, monoclonal, at 1 / 100 dilution), anti - SGLT - 1 (MBL #BMPO22, polyclonal, at 1 / 200 dilution), or anti - SGLT - 2 (Santa Cruz #sc393350, monoclonal, at 1 / 50 dilution) at room temperature for 60 minutes. Diaminobenzidine was used as the substrate for 5 minutes to visualize immunolabeling. Counter - staining was performed with hematoxylin. Erythrocytes present in every section served as internal positive control for GLUT - 1. Liver and intestine tissues were prepared as positive control for GLUT - 2, and SGLT - 1 and SGLT - 2, respectively. PBS was used as negative control instead of the primary antibody on each slide.

Immunohistochemical staining was assessed by both visual observation and computer-assisted cytometrical analysis. Normal parenchyma adjacent tumor was chosen for the analysis. Positively stained tumor cells were counted at x 100 magnification in 5 areas with the strongest staining intensity. Staining intensity was scored into 4 grades as follows: 0 (no staining), 1 (very low), 2 (low), 3 (intermediate), and 4 (high). Intensities of 1 - 2 and 3 - 4 were regarded as weak and strong, respectively. The percentage of positively stained cells was scored into 5 grades as follows: 0 (0%), 1 (< 0 to < 20%), 2 (20 to < 40%), 3 (40 to < 60%), 4 (60 to < 80%), and 5 (80 to 100%). Histological score was calculated as: [sum of intensity (0 to 4) × percentage (0 to 5) in 5 areas] / 5. The visual histological analysis was performed by a single investigator. Immunostained cells were captured as digital images and analyzed in WinROOF (Mitani Corp., Tokyo, Japan) with macroinstructions for analyzing each captured area immunolabeled with chromogen. Image analysis graded the immunostained tumor cells based on staining intensities.

### Statistical analysis

Data with or without normal distribution are expressed as the mean ± S.D. or median (interquartile range), respectively. Statistical analysis for inter - group comparison was performed using the Mann - Whitney U test or Kruskal - Wallis test. Survival curves were drawn using the Kaplan - Meier method and differences in survival were examined using the log - rank test. Spearman's rank correlation coefficient analysis was used to determine the relationship between expressions of glucose transporters. Case distribution was analyzed by Fisher's exact test. Patients were divided into two groups according to pathological parameters, including tumor grade (G 1, 2 vs. G 3), pT (1, 2 vs. 3, 4), pN (0 vs. 1, 2), and pV (0 vs. 1). Receiver operating characteristic (ROC) curves were generated to determine cutoff values that best represented immunostaining intensity. Patients were dichotomized into two groups (low or high) by this cutoff value. The Kaplan - Meier curves were generated to estimate the probability of survival and differences between the curves were assessed by the log rank test. P - values < 0.05 were considered significant. All statistical analyses were performed with EZR software.

## RESULTS

### 

#### Immunostaining

**Figure 1:**
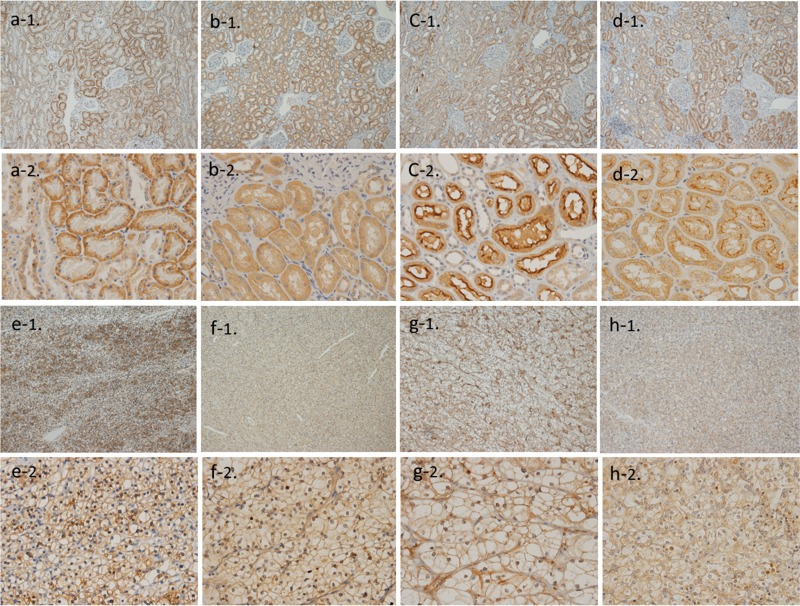
Immunohistochemical staining of paired normal and RCC tissues for GLUT - 1 (a-1, 2, e-1, 2), GLUT - 2 (b-1, 2, f-1, 2), SGLT - 1 (c-1, 2, g-1, 2), and SGLT - 2 (d-1, 2, h-1, 2). Immunostaining was mainly observed at the cell membrane in tumor cells. In the normal parenchyma, staining was detected predominantly at the cell membrane and in the cytoplasm of the proximal and distal renal tubules. Higher - magnification figures indicated the basolateral localization of GLUT and the luminal localization of SGLT. The original magnifications are x 100 (a - 1~h1) and x 400 (a - 2~h - 2).

Immunostaining showed that sugar transporters were predominantly located at the cell membrane and partially in the cytoplasm of tumor cells. In normal parenchyma adjacent to tumor tissue, staining was detected at the cell membrane and in the cytoplasm of proximal and distal renal tubules. Areas containing an abundance of these structures were selected for analyses (Figure-1). Staining intensity distribution in tumor and normal tissues for each transporter is shown in Table-2. The four sugar transporters were consistently present in the normal parenchyma, with a few exceptional cases for SGLT - 1 and SGLT - 2. GLUT - 1 staining in tumor tissue was strong, while GLUT - 2 and SGLT - 1 staining was weak in the majority of cases. A third of the cases lacked SGLT - 2 staining in tumor tissues, and staining in the remaining cases was weak. Staining intensity of GLUT - 1 in tumor tissue was significantly higher than in the normal parenchyma (p < 0.0001). In contrast, normal tissue showed stronger staining for SGLT - 1 (p = 0.0515) and SGLT - 2 (p < 0.001) compared with tumor tissue. Similar staining patterns were observed for GLUT - 2 between tumor and normal tissues.

**Table 2:** Frequency of the sugar transporter expression according to staining intensity.

transporters / intensity	GLUT-1		GLUT-2		SGLT-1		SGLT-2	
	T	N	T	N	T	N	T	N
0	0	0	0	0	0	1 (1.5)	22 (32.4)	2 (2.9)
weak (1,2)	0	37 (54.4)	41 (60.3)	31 (45.6)	46 (67.6)	33 (48.5)	43 (63.2)	34 (50.0)
strong (3,4)	68 (100)	31(45.6)	27 (39.7)	37 (54.4)	22 (32.4)	34 (50.0)	3 (4.4)	32 (47.1)
p-value	<0.0001		0.1218		0.0515		<0.0001	

no. of cases (%)

**T =** tumor; **N =** normal

P-valus were determined by Fisher's exact test.

#### Correlation between pathological parameters and sugar transporter expression

There were strong positive correlations between the degree of immunostaining for each sugar transporter, as determined by subjective counting and objective measurements using image analysis (r = 0.618; p < 0.001 for GLUT - 1, r = 0.674; p < 0.001 for GLUT - 2, r = 0.572; p < 0.001 for SGLT - 1, r = 0.778; p < 0.001 for SGLT - 2). We used the data obtained by image analysis for further evaluations, as it was quantifiable and less subjective. No significant correlations were found between expression of the four sugar transporters and the pathological parameters listed in Table-1 or between any pairs of the four transporters (data not shown).

#### Prognosis according to expression status

**Figure 2:**
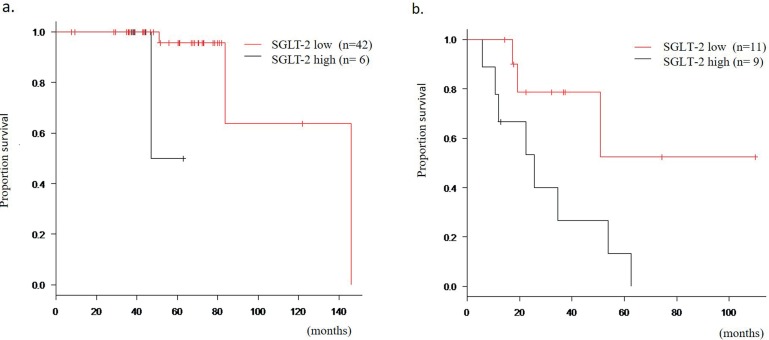
Survival curves according to SGLT - 2 expression in RCC tissue. Patients were divided into two groups (low or high) based on the cutoff value determined by ROC analysis. Patients with higher SGLT - 2 expression showed poorer survival outcomes in both metastatic and non - metastatic disease states. **a)** Overall survival curve according to SGLT - 2 expression in non - metastatic disease; **b)** Overall survival curve according to SGLT - 2 expression in metastatic disease.

During the follow-up, disease progression and death from any cause occurred in 26 (38.2%) and 15 (22.1%) patients, respectively. As shown in Tables 3 and 4, the effect of transporter expression status (low or high) in addition to several pathological parameters on progression free (PFS) and overall survival (OS) were evaluated in non - metastatic and metastatic disease. None of the pathological parameters had prognostic significance. However, SGLT - 2 expression was shown to serve as a prognostic factor in both non - metastatic and metastatic disease in that increased expression of SGLT - 2 in tumor tissue was associated with a shorter OS (Figure-2). Although the univariate analysis isolated only SGLT - 2 expression as a prognosticator, its significance still remained even when all parameters were placed into the multivariate analysis in both non-metastatic (p = 0.049, HR 0.06) and metastatic diseases (p = 0.041, HR 0.25).

**Table 3:** Univariate analysis for various potential prognostic factors in progression free and overall survival in non-metastatic diseases.

	Progression free survival				Overall survival			
	No. pts	HR	95% CI	p-value	No. pts	HR	95% CI	p-value
Nuclear grade(1, 2 vs. 3)	42 / 6	1.02	0.11- 9.17	0.9854	42 / 6	0.39	0.03 - 5.05	0.4719
pT stage (1, 2 vs. 3)	36 / 12	0.29	0.05 - 1.78	0.1669	36 / 12	0.23	0.02 - 2.96	0.2340
pV (0 vs. 1)	18 / 30	1.68	0.34 - 8.35	0.5197	18 / 30	0.98	0.09 - 10.82	0.9875
GLUT-1 (low vs. high)	28 / 20	0.43	0.08 - 2.40	0.3234	10 / 38	0.93	0.08 - 10.25	0.9505
GLUT-2 (low vs. high)	25 / 23	1.38	0.23 - 8.42	0.2244	25 / 23	0.56	0.03 - 8.98	0.6771
SGLT-1 (low vs. high)	12 / 36	3.03	0.51 - 18.14	0.2069	12 / 36	2.05	0.18 - 23.89	0.5578
SGLT-2 (low vs. high)	41 / 7	3.45	0.63 - 18.92	0.1355	42 / 6	0.06	0.004 - 0.99	0.0081

**HR =** hazard ratio; **95%CI =** 95% confidential interval

**Table 4:** Univariate analysis for various potential prognostic factors in overall survival in metastatic diseases.

	No. pts	HR	95% CI	p-value
Nuclear grade (1, 2 vs. 3)	11 / 9	0.70	0.21 - 2.31	0.5593
pT stage (1, 2 vs. 3, 4)	9 / 11	0.40	0.10 - 1.51	0.1615
pV (0 vs. 1)	3 / 17	2.33	0.29 - 18.42	0.4104
pN (0 vs. 1, 2)	13 / 7	2.06	0.62 - 6.89	0.2303
GLUT-1 (low vs. high)	9 / 11	2.29	0.66 - 7.94	0.1784
GLUT-2 (low vs. high)	7 / 13	0.97	0.29 - 3.22	0.9630
SGLT-1 (low vs. high)	4 / 16	3.02	0.84 - 10.83	0.0756
SGLT-2 (low vs. high)	11 / 9	0.25	0.07 - 0.95	0.0280

**HR =** hazard ratio; **95%CI =** 95% confidential interval

## DISCUSSION

Increased glucose uptake is a key alteration associated with the high glycolytic rate in cancer cells, and reprogramming energy metabolism is considered one of the “hallmarks of cancer”. A representative mechanism of glucose uptake into cells is facilitative diffusion mediated by GLUTs. GLUT - 1 overexpression is well documented in cancer and is often associated with a poor prognosis (8). Elevated GLUT - 1 expression was also demonstrated in RCC tissue, but its association with pathological parameters or prognosis has not been determined (9-12). Acceleration of glucose accumulation via GLUTs in cancer is the basis for clinical detection and staging by positron - emission tomography using (^18^F) fluorodeoxy - glucose (^18^F - FDG PET) (22). However, ^18^F - FDG PET does not appropriately detect some types of cancers, including RCC, and its use for diagnosis and staging is not currently recommended in routine clinical practice (23). This may indicate that another class of glucose transporters not detected by ^18^F - FDG PET, such as SGLTs, could contribute to glucose utilization in these cancers. In fact, SGLT - 1 and SGLT - 2 are expressed in several types of cancer and may be involved in glucose transportation in tumor tissue (13-19).

The present study demonstrated constitutive expression of four sugar transporters in the renal tubules by immunostaining. It has been demonstrated that diabetic patients may have their transporter patterns different from non - diabetics. According to the report based on the animal model, long - term DM condition resulted in significant increase in GLUT - 1 level in the renal proximal tubules (23). On the other hand, it has been reported that in whole renal tissue obtained from human subjects with DM and non - DM, expressions of SGLT - 1 / GLUT - 1 and SGLT - 2 / GLUT - 2 are coupled and slightly lower in DM patients as compared with well - matched people without DM (24). Another study using human renal biopsy specimens showed that the expression of SGLT - 1 mRNA was markedly increased, while the level of GLUT - 2 and SGLT - 2 mRNA are downregulated in the kidney of diabetic patients (25). Thus, the expression status of GLUT - 1 / GLUT - 2 and SGLT - 1 / SGLT - 2 in diabetic patients seems inconsistent compared with non - diabetic counterparts among the studies. In the present study, expressions of the four sugar transporters in DM patients did not differ from those in non - DM patients (data not shown).

SGLT - 1 is highly expressed in the gastrointestinal tract and is the major transporter of glucose and galactose. SGLT - 1 is also expressed in the liver, lung, and kidney, while SGLT - 2 is selectively expressed in the kidney (26). Phlorizin, a non - selective but potent inhibitor of SGLT that also inhibits both renal reabsorption and intestinal absorption of glucose, has antihyperglycemic effects, but is accompanied by severe gastrointestinal symptoms (27). Selective SGLT - 2 inhibitors that block glucose reabsorption in the renal proximal tubules have recently been developed as antidiabetics (28). Given that SGLT - 1 and SGLT - 2 are expressed in various tumors, drugs that inhibit these transporters may be successfully used for anticancer therapy. Such clinical background information prompted us to investigate the expression patterns of GLUT - 1 and GLUT - 2 as well as SGLT - 1 and SGLT - 2 in RCC tissue, which are also expressed in the normal kidney, and to further evaluate possible associations with pathological parameters and prognostic values.

There have been several studies on the GLUTs expression in RCC. Nagase et al. demonstrated that immunohistochemical localization of GLUT - 1 was observed at the plasma membrane in RCC cells in most cases (84.6%), while several cases (5.3%) showed positive staining in renal tubules. Positive immunostaining in RCC cells has only been observed in clear cells, but not in spindle or granular cells (9). Immunohistochemical GLUT - 1 expression patterns have also been investigated in association with hypoxia inducible factor - 1α (HIF-1α). Most patients with clear cell RCC have high GLUT - 1 staining and a significant correlation with HIF - 1α, suggesting the role of GLUT - 1 as a rapid energy deliverer under HIF-1α control to maintain the proliferative characteristics of tumors (10). Comprehensive immunohistochemical evaluation of GLUT - 1 expression was conducted using 228 RCC samples. A vast majority (86.2%) of clear cell RCC showed strong, diffuse staining of tumor cells in a membranous pattern, while the minority of other RCC types had weak staining with a cytoplasmic pattern (11). Suganuma et al. examined GLUT gene expression at the mRNA level and showed higher GLUT - 1 but lower GLUT - 4, 9, and 12 expression in clear cell RCC tissues compared with normal tissue (12). GLUT - 2 levels were not different between normal and RCC tissues. Despite different methods of detection, GLUT - 1 and GLUT - 2 expression patterns in normal and tumor tissues were similar to our results. A recent exhaustive meta - analysis demonstrated that GLUT - 1 overexpression was associated with poor survival in most solid tumors (8), although GLUT - 1 status appears to be unrelated to pathological factors or prognosis of patients with RCC according to limited data (10, 11). Similarly, both GLUT - 1 and GLUT - 2 expression was unrelated to any pathological variables as well as patient survival in the present study.

Data regarding SGLT expression in cancer are scarce. A pioneer study demonstrated that sodium - dependent sugar cotransporter expression in HT29 colon cancer cells was modulated by addition or deprivation of glucose in culture medium (29). The first study measuring SGLT - 1 and SGLT - 2 gene expression in human tissue was performed using autopsied samples from normal lung and primary lung tumors together with their metastatic lesions. SGLT - 1 and SGLT - 2 expression was similar between lung cancer and paired normal tissue. SGLT - 2 expression was significantly higher in metastases than in primary tumors, while SGLT - 1 expression was unchanged, suggesting that SGLT - 2 plays a role in glucose uptake in metastatic lesions in lung cancer (13). SGLT - 1 expression, but not SGLT - 2, was observed in short - term cultures of head and neck squamous cell carcinoma (HNSCC) by RT - PCR. SGLT - 1 mRNA expression preferentially occurred in cultures originating from moderately and well differentiated tumor cells. SGLT - 1 immunostaining was also restricted to differentiated tumor tissue, indicating differentiation - dependent SGLT - 1 expression in HNSCC (14).

Although SGLT is overexpressed in tumor tissue, its clinical significance has yet to be established. In pancreatic adenocarcinoma, high SGLT - 1 expression was significantly associated with longer disease - free survival and better overall survival (15). Likewise, high SGLT - 1 levels were associated with improved survival after chemo - radiotherapy in patients with cervical cancer (16). In contrast, SGLT - 1 expression was positively associated with tumor stage, and SGLT - 1 overexpression was an independent biomarker for poor prognosis in patients with ovarian cancer (17). SGLT - 1 levels had no impact on prognosis, although overexpression was related to advanced clinical stages in colorectal cancer (18). In the present study, neither SGLT - 1 nor SGLT - 2 expression was related to pathological variables in RCC tissue. Moreover, SGLT - 1 expression status was not associated with patient prognosis. SGLT - 2 protein levels successfully predicted survival for patients with RCC, regardless of metastasis. None of the pathological parameters were determined to have prognostic significance in both non - metastatic and metastatic disease. Because metastasis was the only remaining independent prognostic factor in whole patients by multivariate analysis (data not shown), the prognostic power of pathological variables was lost when analyzed separately based on metastatic status. We propose that increased SGLT - 2 expression is associated with a poor prognosis in clear cell RCC. This may support a promising therapeutic strategy against RCC using agents that target SGLT - 2. Scafoglio et al. demonstrated functional expression of SGLT - 2 using SGLT - specific α - methyl - 4 - deoxy - 4 - ^18^FDG in pancreatic and prostate cancers. Functional activity was blocked by a specific SGLT - 2 inhibitor, dapagliflozin, which is clinically used as antidiabetics (20). While this approach may be applicable to RCC, it is beyond the scope of this study and should be investigated in future research.

The present study has several limitations. The retrospective nature of the study and a small number of patients are major limitations. Given the small subset of patients, the results may be considered suggestive and hypothesis - generating rather than conclusive. Thus, future confirmatory studies will be necessary to define the findings. In addition, despite our efforts to select representative areas in the tumor tissue, intra - tumor heterogeneity is an unavoidable limitation in studies using archival RCC samples.

## CONCLUSIONS

We for the first time investigated SGLT expression by immunostaining in RCC tissue and raised the possibility that increased SGLT - 2 expression is associated with unfavorable outcomes. The prognostic implications of SGLT - 2 suggests that SGLT - 2 inhibitors may be useful in treating RCC. In tumors expressing SGLT - 2, potent selective SGLT - 2 inhibitors may decrease glucose uptake, disrupt glycolysis, and suppress tumor growth without harmful side effects, as they are already used to safely treat diabetes. These issues deserve further investigation to pave the way for novel therapeutic approaches against RCC.

## ABBREVIATIONS

GLUTs: = glucose transporters.
SGLTs: = sodium - dependent glucose transporters
RCC: = renal cell carcinoma
^18^F - FDG: = [^18^F] fluorodeoxy - glucose
PFS: = progression free survival
OS: = overall survival
